# Evaluation of Central and Peripheral Fatigue in the Quadriceps Using Fractal Dimension and Conduction Velocity in Young Females

**DOI:** 10.1371/journal.pone.0123921

**Published:** 2015-04-16

**Authors:** Matteo Beretta-Piccoli, Giuseppe D’Antona, Marco Barbero, Beth Fisher, Christina M. Dieli-Conwright, Ron Clijsen, Corrado Cescon

**Affiliations:** 1 Rehabilitation Research Laboratory, Department of Business Economics, Health and Social Care, University of Applied Sciences and Arts of Southern Switzerland, SUPSI, Manno, Switzerland; 2 Department of Molecular Medicine and Sport Medicine Centre Voghera, University of Pavia, Pavia, Italy; 3 Division of Biokinesiology and Physical Therapy, University of Southern California, Los Angeles, United States of America; 4 Department of Business Economics, Health and Social Care, University of Applied Sciences and Arts of Southern Switzerland, SUPSI, Landquart, Switzerland; University of Jaén, SPAIN

## Abstract

**Purpose:**

Over the past decade, linear and non-linear surface electromyography descriptors for central and peripheral components of fatigue have been developed. In the current study, we tested fractal dimension (FD) and conduction velocity (CV) as myoelectric descriptors of central and peripheral fatigue, respectively. To this aim, we analyzed FD and CV slopes during sustained fatiguing contractions of the quadriceps femoris in healthy humans.

**Methods:**

A total of 29 recreationally active women (mean age±standard deviation: 24±4 years) and two female elite athletes (one power athlete, age 24 and one endurance athlete, age 30 years) performed two knee extensions: (1) at 20% maximal voluntary contraction (MVC) for 30 s, and (2) at 60% MVC held until exhaustion. Surface EMG signals were detected from the vastus lateralis and vastus medialis using bidimensional arrays.

**Results:**

Central and peripheral fatigue were described as decreases in FD and CV, respectively. A positive correlation between FD and CV (R=0.51, p<0.01) was found during the sustained 60% MVC, probably as a result of simultaneous motor unit synchronization and a decrease in muscle fiber CV during the fatiguing task.

**Conclusions:**

Central and peripheral fatigue can be described as changes in FD and CV, at least in young, healthy women. The significant correlation between FD and CV observed at 60% MVC suggests that a mutual interaction between central and peripheral fatigue can arise during submaximal isometric contractions.

## Introduction

Fatigue is a psychophysiological state experienced in daily life. It can be described as a feeling of weakness or muscle pain, or a decrease in performance during physical or cognitive activities [1]. When considered at the level of skeletal muscle, fatigue can be defined as any reduction in the maximal capacity to generate force or power output induced by exercise [2,3] and represents a common and major limiting factor in sports performance. If muscle contraction is fundamental to unraveling muscle fatigue, chronic exercise training enhances the ability of the muscles to resist fatigue [4].

Muscle fatigue is also a fundamental symptom in a wide range of pathological conditions, including neurological (e.g., Parkinson’s disease, multiple sclerosis, stroke) and non-neurological (e.g., cancer, metabolic disorders, and cardiac, muscle, and respiratory conditions) diseases. Because muscle fatigue impacts so many disorders, leading to functional limitations and decreased quality of life, a critical first step in developing effective interventions is to fully understand the mechanisms underlying muscle fatigue and how it can be measured.

Multiple processes contribute to muscle fatigue, and many of these appear soon after the beginning of a voluntary contraction. Fatigue continues to develop throughout the contraction, and starts to remit when the task is complete [5]. Although skeletal muscle manifests fatigue during a sustained task, the origin of fatigue is not exclusively muscular. In fact, muscle fatigue develops in different parts of the body and can be divided into central and peripheral fatigue according to its origin [6]. During a sustained maximal voluntary contraction (MVC), healthy subjects develop both central and peripheral aspects of fatigue [7,8]. Central fatigue can originate from any structure above the neuromuscular junction, from the central nervous system to the peripheral nerves and might result from a combination of intrinsic motorneuronal properties, reflex inhibition and disfacilitation, Renshaw cell inhibition and insufficient drive from supraspinal sites [9,10], for example due to decreased subject motivation [11]. Central fatigue may occur during sustained maximal contractions, fatiguing exercises, and disease states in which there is disruption of upper motor neuron function [12]. Peripheral fatigue reflects local changes in the muscle (e.g., decreased calcium release form the sarcoplasmic reticulum, increased concentration of inorganic phosphate and transient large increase in ADP concentration) and hampers the execution of descending central commands [13,14]. Peripheral fatigue results also in a decreased muscle fiber conduction velocity (CV) [15,16], related to a decrease of the intracellular pH [17–19].

A fundamental issue with respect to understanding muscle fatigue is its assessment. The use of the twitch interpolation technique is widely used and is considered the most reliable method to estimate the origin of neuromuscular fatigue [3,11,12,20]. Peripheral fatigue is generally measured by comparing the force responses to electrical stimulation before and after a fatiguing exercise, whereas to determine the contribution of central factors to fatigue, several studies have used variants of the twitch interpolation technique, which consist in superimposing single twitches or high-frequency doublets on a MVC and to compare the superimposed response to the potentiated response obtained from the relaxed muscle (for a review, see [21]).

To overcome the twitch interpolation technique limitations (e.g. discomfort from stimulation, impossibility to test the neuromuscular function in physiological situations, contribution of intramuscular processes to superimposed force with fatigue [2,22]), the evaluation of central and peripheral components of fatigue might be explored using indexes based on mechanical variables, such as force or torque, or associated with the surface electromyography (sEMG) signal [23].

The principal issue with sEMG-based indexes is their lack of sensitivity in differentiating between central and peripheral aspects of fatigue; therefore, a number of more sensitive parameters have been developed to study sEMG signals during isometric and dynamic fatiguing contractions (for an exhaustive and comprehensive review, see [24]).

Estimation of the muscle fiber conduction velocity (CV) slope during an isometric task is the most robust index of peripheral fatigue [25–31] and, if the MU pool is stable, this variable correlates with fiber size and type [32,33].

Fractal dimension (FD) is an index based on the assumption that the normal sEMG interference pattern has fractal properties [34]. FD was initially used to characterize levels of muscle activation [35,36] and patterns of MU recruitment [37], but Mesin and colleagues [38] later suggested that FD is also related to MU synchronization during muscle fatigue. Decreases in FD may be considered as indicators of progressive MU synchronization.

However, as with other non-linear procedures such as recurrent quantification analysis, percentage of determinism, Dimitrov’s spectral fatigue index (FInsm5), and entropy [39–42], it is difficult to relate these parameters to physiological changes in muscle properties resulting from muscle fatigue. Mesin and colleagues [38] compared FD with other muscle fatigue indexes computed from both synthetic sEMG and experimental signals. They found that FD was the index least affected by CV changes, weakly affected by fat layer thickness and most related to the level of synchronization, suggesting that FD could be a promising index of central fatigue. Troiano et al. [43] found that the FD obtained during an isometric contraction at 50% MVC decreased, indicating that the synchronization level increases with muscle fatigue, although the relation between FD and force level remains controversial [34,35,38].

Hence, the objectives of this study were: (1) to describe myoelectric manifestations of fatigue using FD and CV slopes as indices of central and peripheral fatigue, respectively, during isometric contractions in vastus medialis (VM) and vastus lateralis (VL) muscles in healthy subjects; and (2) to analyze the relationship between FD and CV slopes and the correlation of the FD slope with another indicator of central fatigue, i.e., voluntary activation deficit (VAD.

## Methods

### Subjects

The study was approved by the local ethics committee of the Swiss Italian Health and Sociality Department, Switzerland. All procedures were conducted according to the Declaration of Helsinki. All participants signed a written informed consent form before participation in the experiments. Twenty-nine healthy female volunteers (mean±standard deviation: age 23±3 years; height 167±6 cm; weight 62±9 kg) from a university setting were recruited to participate in the study. The women were moderately active (≥3 days of moderate trainings per week) and had no previous injuries of the lower limbs.

In addition, two healthy female elite athletes were recruited to the study in order to analyze their behavior during a fatiguing task: a power athlete (age 24 years) whose specialties were the javelin and sprinting, and an endurance athlete (age 30 years) whose specialties were long-distance running and triathlon.

### Electrical stimulation

VAD was evaluated from electrically elicited contractions using the twitch interpolation method [44–46]. Electrical nerve stimulation was delivered over the femoral nerve trunk using 100 μs square-wave pulses from a single-channel constant-current stimulator (DS7AH; Digitimer, Welwyn Garden City, UK) with a custom-made on/off switch. Stimulation was delivered through an electrode pouch with a 4 mm socket and press-stud button (Meditech, Polo di Torrile, Italy) as the anode (120×80 mm), positioned on the skin at the gluteal fold. An adhesive electrode in a monopolar arrangement was used as the cathode (35×45 mm; Spes Medica, Battipaglia, Italy) and was placed on the skin of the femoral triangle. The electrode positions and pulse duration of 100 μs were chosen to reduce perceived discomfort during electrical stimulation of the femoral nerve.

### EMG and force measurements

**Fig 1:**
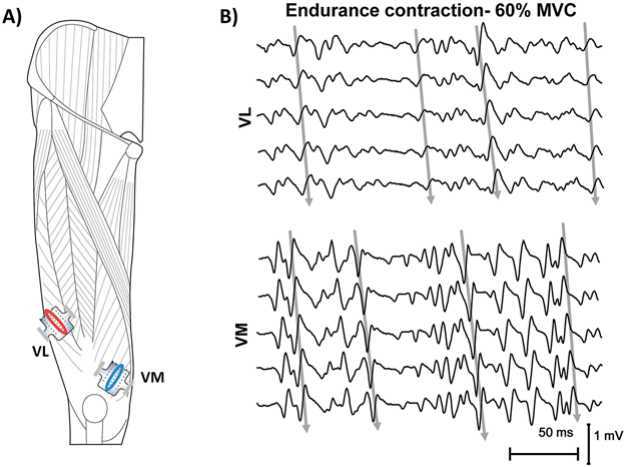
Electrode arrays positions on VL and VM muscles (A) and representation of the EMG signals (B). Myoelectric signals were detected in single differential configuration, using bidimensional arrays, positioned along the length of the muscles, between the innervation zone and the distal tendon. Channels chosen by visual analysis, for the subsequent global analysis, are indicated by the ovals.

Myoelectric signals were detected from the VL and VM in a single differential configuration using two bidimensional arrays of 30 electrodes (3 mm diameter, 6x5 grid, 8 mm interelectrode distance; Spes Medica) (Fig 1). These muscles were chosen primarily in order to obtain high-quality sEMG signals, as previously described [47], but also because they are easily accessible for sEMG measurements. The adhesive arrays were applied between the innervation zone and the distal tendon on the VL and VM muscles, identified with a dry linear array as previously described [48]. The EMG signals were amplified (EMG-USB2; OT Bioelettronica, Turin, Italy), band-pass filtered (10–750 Hz), sampled at 2048 Hz, and stored on a computer.

A custom-developed ergometer (SUPSI; OT Bioelettronica) was used to measure knee torque with a torque meter operating linearly in the range 0–1000 Nm. The torque signal was amplified (MISO II; OT Bioelettronica) and stored on a computer with the sEMG data. The torque signal was displayed on a screen, providing real-time biofeedback.

### Experimental procedure

Participants sat on an ergometer chair with the knee flexed at 120°, the pelvis and thigh firmly strapped to the plinth with a seatbelt, and the leg fixed to the ergometer with a strap attached to the chair, 2–3 cm above the lateral malleolus.

After placement of the stimulation electrodes, the stimulation intensity required for maximum torque twitch was determined prior to the experimental session by increasing the amperage until the torque twitch reached a plateau. The EMG electrodes were then applied to the VL and VM muscles.

After 5 min rest, four isometric MVCs of 2–3 s were performed, each separated by 2 min rest. During each contraction, the force trace was displayed to participants on a computer monitor as visual feedback. Participants were instructed to increase the force up to the maximum, and to hold it as steady as possible. Participants were given verbal encouragement.

During the third and fourth maximal efforts, a supramaximal doublet (two rectangular pulses of 100 μs duration, approximately 300–600 mA, with an interval of 10 ms in between) was delivered to the femoral nerve. The first doublet was delivered about 1 s after the peak force. The second doublet was delivered to the relaxed muscle 2–5 s after the superimposed twitch to evoke a “control” twitch potentiated by the previous contraction.

Next, a low-level contraction (20% MVC) was performed for 30 s, which was used as a negative control of muscle fatigue after which the subjects were asked to provide a value on a visual Borg scale, ranging from 6 to 20 [49]. Eventually, the subjects had to perform a high-level contraction (60% MVC) maintained until exhaustion, during which they were verbally encouraged to keep the force level for as long as possible, until the force value decreased to below 90% of the target (endurance time, i.e. the time for which a subject is able to maintain the requested mechanical task [50]). Participants had a 5 min rest between contractions.

### Signal processing

FD was computed using a numerical algorithm [34] with non-overlapping signal epochs of 1 s, where the EMG signals were supposed to be stationary. The FD of a continuous-time signal takes values between 1 (smooth signals) and 2 (stochastic or deterministic signals filling the whole space) [38].

CV was estimated using a multichannel algorithm [51] on single differential signals. CV values outside the physiological range (2–8 m/s) were excluded from the analysis.

A supramaximal doublet was delivered during MVC and during rest. VAD was calculated as the ratio between the torque produced by the superimposition of the supramaximal twitch on a peak isometric contraction (St) and the torque produced by the same stimulus in the potentiated, resting muscle (Rt) [44]:

Voluntary Activation Deficit (%)= (St/Rt)*100

### Statistical analysis

Linear regression over time was applied to FD and CV in order to extract an initial value and slope. Only the first 25 s were considered to compute the regression line and the corresponding initial values and slopes, in order to use the same duration for all signals.

Wilcoxon signed-rank paired tests were used to compare the initial values of FD and CV at 20% and 60% MVC for both the vasti muscles, using SPSS (IBM, Armonk, NY, USA). In addition, the same analysis was performed to compare the initial values of FD and CV between the VL and VM muscles. Statistical significance was set to α = 0.05.

Correlations between FD, CV, and VAD during the high-level contraction were tested using Pearson’s correlation coefficient (R).

Statistical analysis was performed with data from the recreationally active participants, excluding the two elite athletes, in order to have a homogeneous group. Values from the elite athletes are included in the graphs to show their behavior compared with that of the other participants.

Results are reported as median, interquartile range and range.

## Results

Table 1 summarizes the results for FD and CV at 20% and 60% MVC, in the VM and VL muscles. Initial values of FD and CV were significantly higher at 60% MVC than at 20% MVC for both the VL and VM muscles (p<0.001). Initial estimates of CV were significantly higher for the VL compared with VM muscle at both 20% and 60% MVC (p<0.001). The initial values of FD were significantly lower for the VL compared with VM muscle at both 20% and 60% MVC (p<0.001).

**Table 1:** Initial values and normalized slopes of FD and CV for VL and VM muscles at 20 and 60% MVC. Values are indicated as median (Interquartile Range).

		20% MVC		60% MVC	
		VL	VM	VL	VM
**Fractal dimension (FD)**	**Initial**	1.5449 (0.0350)	1.5515 (0.0247)	1.5659 (0.0343)	1.5869 (0.0388)
	**Slope (%/s)**	-0.0250 (0.0438)	-0.0034 (0.0394)	-0.0517 (0.0655)	-0.0288 (0.0790)
**Conduction velocity (CV)**	**Initial (m/s)**	7.0408 (1.4123)	5.0848 (0.7080)	7.2778 (0.7922)	6.0731 (1.7102)
	**Slope (%/s)**	-0.1392 (0.2318)	0.0007 (0.1567)	-0.1172 (0.3240)	-0.1574 (0.1562)

**Fig 2:**
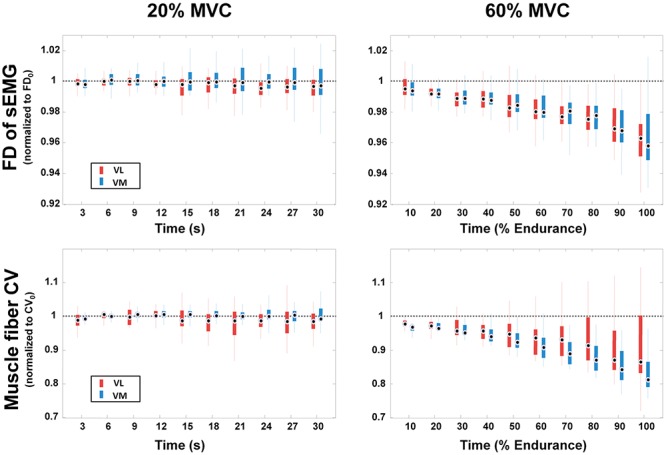
Time course of FD and CV in the VL (red) and VM (blue) muscles during 20% and 60% MVC. Data are presented as median (circles), interquartile range (rectangles), and range (vertical lines) normalized with respect to their initial values. The time axis is divided in ten epochs. For the 20%MVC the epoch length is 3 s, being 30 s the duration of the contraction, while for the endurance 60%MVC contraction the epoch length is 10% of the total endurance time.

The average time course of FD and CV during 20% and 60% MVC is shown in Fig 2. Significant negative slopes for FD and CV were observed during the sustained 60% MVC compared with the lower-intensity contraction, in particular in the VM muscle.

At 20% MCV contraction the lack of FD and CV change over time was paralleled by an average Borg scale score of 10.8 ± 2.1, indicating a fairly light perceived exertion whereas at 60% MVC the average score was 18.9 ± 1.4, indicating a very, very hard perceived exertion.

**Fig 3:**
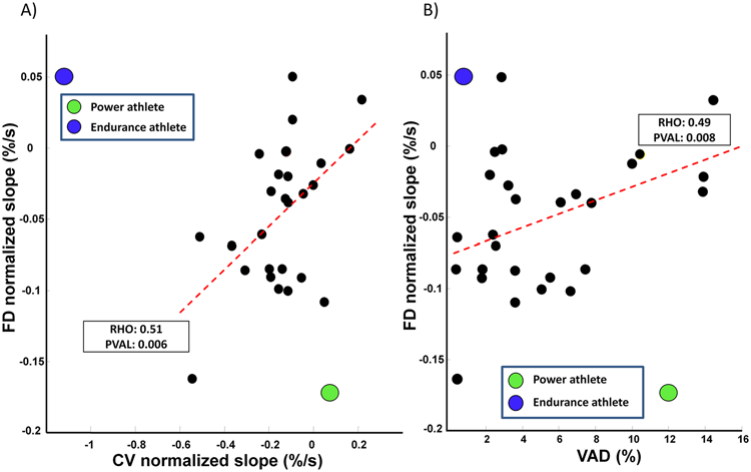
Scatter plot of the normalized slopes of (A) FD versus CV and (B) FD versus VAD during 60% MVC. Data from the power and endurance athletes are superimposed in different colors. The R and p values of the Pearson’s correlation coefficient are indicated. Dashed red lines indicate linear regression performed on the data from healthy subjects.

A significant positive correlation was observed between FD and CV (R = 0.52, p<0.01; Fig 3A) and between VAD and FD (R = 0.49, p<0.01; Fig 3B) during 60% MVC.

**Fig 4:**
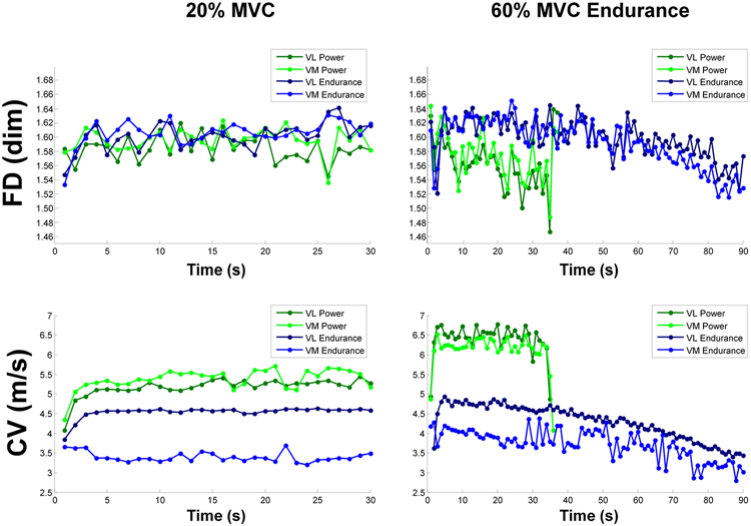
Time course of FD and CV in the VL and VM muscles of the power and endurance athletes during 20% and 60% MVC.

Fig 4 shows the time course of FD and CV of the VM and VL muscles in the elite athletes. At 60% MVC a negative slope was found in both muscles for FD, unlike CV, in the power athlete whereas a negative slope was found for CV, unlike FD in the endurance athlete.

The distribution of torque exerted by the healthy subjects was 218 ± 68 Nm, while the distribution of endurance time was 57.9 ± 12.1 s, while the torque exerted by the elite athletes was 453 Nm for the power athlete and 273 Nm for the endurance athlete, with endurance times of 33 and 89 s, respectively.

## Discussion

### Peripheral fatigue

It is widely accepted that increasing the force output results in progressively higher CV [52], with this phenomenon stopping at different thresholds below MVC depending on the muscle and the type of contraction [53]. Accordingly, in the VM and VL muscles during isometric knee-extension contractions at 60% MVC, the initial value of muscle fiber CV was higher than at 20% MVC. This phenomenon might be explained by the recruitment of progressively larger MUs with increasing force output, and therefore with progressively higher CV values during fatiguing contractions, according to the Henneman size principle [54].

In agreement with previously published data [55,56], at both force levels analyzed the initial value of CV was greater in the VL than the VM muscle. It is possible that dissimilar phenotypic features of the muscles analyzed might contribute to the observed differences in CV between muscles. In particular, although no data are available on differences in fiber size between vasti muscles, the known interrelationship between fiber size, fiber composition, and CV might have an influence [57].

In fact, muscle fiber composition and size have been shown to influence the EMG signal during fatigue. For example, a more pronounced decrease in several spectral parameters and CV has been observed in muscles with a greater percentage of type II fibers [33,58,59]. Thus far, the vasti muscles can be differentiated based on their proportions of fiber types: biopsy studies have demonstrated a significantly lower proportion of type II fibers in VM compared with VL muscles [60–65]. Therefore, we can hypothesize that the behavior of central and peripheral fatigue, estimated by FD and CV, could be different in the VM and VL muscles.

However, initial values of EMG variable estimates are not only related to the recruited MU pool, but are also affected by factors such as subcutaneous tissue thickness, skinfold thickness and fiber end effects [66]. In women, the VL is covered by a thicker skinfold, compared with men [60] and it is reasonable to expect that the iliotibial band overlying the VL could alter EMG estimates, such that the initial value of CV would be elevated (due to an increased fiber end effect).

We observed a change in the CV slope at 60% MVC, in accordance with previous published papers which highlighted a strict correlation between peripheral fatigue and CV changes over time during sustained isometric contractions as a result of increased metabolites [13, 28, 67, 68]. The lack of change in the CV slope at 20% MVC suggested the absence of significant peripheral fatigue at low force output, and identified this force output as a not fatiguing task in the muscles tested in our subjects. Interestingly, the Borg score reported by the subjects paralleled the observed changes in CV (and FD), thus confirming the significance of both force levels in terms of perceived fatigue. Moreover, data on CV, although not allowing to identify the lower limit and the time course of peripheral fatigue, identify this limit above 20% MVC, and put forward that this task extended for a short period of time may be considered a useful target to study the abnormal peripheral response to sustained contractions both in physiological and pathological conditions.

### Central fatigue

The initial value of FD was higher in VM compared with VL muscle at both 20% and 60% MVC, suggesting that the initial status of MU synchronization is lower in the VM. This phenomenon could also be explained by the thicker subcutaneous tissues overlaying the VL muscle acting as low-pass filters, possibly reducing the interference level of the EMG signals [69]. When the requested effort was 60% of the MVC, the initial value of FD was statistically higher than with the low-intensity contraction, potentially due to the recruitment of additional MUs. The negative slopes of FD suggest an increase in MU synchronization during the endurance 60% MVC, probably as a result of an adaptation to muscle fatigue by the central nervous system. Interestingly, at 20% MVC the level of synchronization remained almost constant, just as with the muscle fiber CV (Fig 2). During the endurance contraction, the FD slope was significantly different between the two vasti muscles (p<0.05), suggesting a higher degree of fatigability of the VL versus the VM muscle, as already indicated by the CV slope.

Importantly, data obtained on 60% MVC of the VM, that described simultaneous changes in CV and FD over time, suggest that this contraction level cannot be considered a useful task to identify whether the change in CV may precede or follow that of FD and that this force level (if any) should be searched between 20% and 60% MVC in this muscle.

Overall these observations support the hypothesis that central fatigue during a specific task may differ not only among muscles [70–72]{Zijdewind, 2001 #923} but also among different muscle bellies of the same muscle (i.e. VM and VL in the quadriceps femoris), and may be related to different late adaptations to muscle fatigue between the two vasti muscles. In fact, differences in late adaptation of the neural discharge during sustained contractions may correlate with different impulse rates at the beginning of the discharge, and thus with MU distribution [73].

Indeed, the presence of a significantly lower proportion of type II fibers in VM versus VL muscles [65] suggests the presence of a higher percentage of slow-twitch MUs with a lower mean firing rate than that of fast-twitch MUs [74]. Thus, when larger MUs in the VL muscle are synchronized as a result of muscle fatigue, the sEMG signal is less interferent and thus has lower values of FD, while when smaller MUs in the VM are synchronized, the sEMG has the same level of interference.

### Correlations between CV slope, FD slope, and VAD

A significant correlation was observed between the normalized slopes of CV and FD in the group of recreationally active participants. This might be explained by the fact that the fatiguing task may induce both central and peripheral fatigue in these healthy, recreationally active women, as a result of mutual interactions between central and peripheral mechanisms [75].

Above all, a significant direct correlation between FD slope and VAD (R = 0.49, p<0.01) was observed. Considering that a negative slope of FD can be interpreted as an increase of MU synchronization, whereas VAD indicates the whole central activation capacity (spatial recruitment, temporal recruitment, and MU synchronization) and the failure of the central nervous system to recruit all of the muscle fibers during a MVC, our results put forward that FD and VAD may mirror different aspects of central fatigue. In addition, VAD is a component of central fatigue that can be measured before a fatiguing task, and represents a sort of baseline deficit of muscle activation, while the slope of FD is an index of central fatigue, measured during a fatiguing endurance task. Thus, these two variables reflect different aspects of central fatigue.

However, VAD might be also the result of a decrease in motor neuron firing rates rather than a reduction in the extent of MU recruitment [76], or of different muscle size.

During fatiguing contractions, the central drive to a muscle has to increase, leading to synaptic input that is common to more than one neuron. As known, muscle fatigue has been described in terms of motor unit recruitment patterns [77]; with the onset of localized muscle fatigue, the increased central drive leads to increased synchronization of motor units firing patterns, and this might be explained by the commonality in the pre-synaptic input to motor units [78].

Indeed, the more MUs are synchronized, the more the raw signals present larger peaks and appear to have less interference, which brings about a decrease of FD slope.

### Elite athletes

We analyzed the behavior of two elite athletes, a power athlete and an endurance athlete, in order to show a probable different behavior of the variables and how their rate of change can highlight different strategies of adaptation to fatigue. These athletes were considered as case studies and were compared with the group of recreationally active women.

The torque exerted by the power athlete was much higher than the average torque of healthy subjects, while the endurance athlete had a torque comparable to the baseline subjects. On the other hand, the endurance times of the two athletes were located on the opposite sides of the time distribution of baseline subjects, the endurance athlete higher and the power athlete lower respectively, as expected.

The two athletes showed an opposite behavior: a negative slope was found for FD, unlike CV, in the power athlete whereas, on the other hand, a negative slope was found for CV, unlike FD in the endurance athlete. Interestingly, when superimposed with the other values, the negative relationship between [FD *vs* CV] and [FD *vs* VAD] normalized slopes found in the elite athletes, showed an opposite behavior being higher in the endurance athlete and lower in the power athlete and appeared uncorrelated with those of the moderately trained individuals (Fig 3A and 3B).

Interestingly, while the recreationally active participants generally exhibited both central and peripheral fatigue, the endurance athlete showed only peripheral fatigue with an unchanged level of synchronization, and the power athlete showed central fatigue (both of VAD and FD slope absolute values) without CV changes during the fatiguing task (Figs 3 and 4). Although it cannot be known whether the fatigue behaviors observed in the two elite athletes are representative of the entire populations of power and endurance athletes, their CV and FD slopes might reflect differences in training-induced adaptations of the central nervous system to fatigue and may be helpful in encouraging scientists to plan future research in this field.

### Limitations

The limitations of this study are mainly related to technical constraints. First, we selected only women, for convenience, as women comprised the majority of volunteers for this experiment. Muscle fiber composition and central mechanisms related to fatigue might be different in men, as demonstrated by sex-related differences in muscle fatigue during isometric fatiguing contractions, as observed for knee extensors [79–84]{Russ, 2003 #1002}{Hunter, 2009 #987}{Hunter, 2009 #987}. Furthermore, while men are generally stronger than women, there is accumulating evidence that women are better at sustaining continuous muscle contraction at low to moderate intensities [85–87]. It is therefore reasonable to hypothesize that the behavior of the FD and CV slopes could be slightly different in men.

Second, we investigated only two muscles, which, of course, do not represent the behavior of the entire leg.

Third, the experimental protocol did not allow us to differentiate between the sources of muscle fatigue during contractions. From a physiological point of view, the mutual interaction of central and peripheral fatigue mechanisms does not, as far as we know, allow separation of the two systems. Artificial stimulus techniques, such as electrical or magnetic stimulation, despite having some level of discomfort, are likely to remain the only methods of selectively assessing central and peripheral fatigue (for an example, see [70]). Furthermore, whether the interpolated twitch provides a valid measure of VAD is still debated [88,89], but authors agree that it helps in the detection of altered drive to muscles, for instance with fatigue [2,21].

Another limitation is the choice of VL muscle. The subcutaneous layer and the pennation angle of the muscle fibers made the recording of EMG signal difficult, especially the estimation of CV. indeed the higher variance in the CV estimation in VL muscle with respect to VM muscle was probably due to non-optimal electrode positioning on this muscle.

Finally, in recent years alternative descriptors of MU synchronization, with low dependency on muscle fiber CV (e.g., sub-band skewness, Piper rhythm), have been developed and tested [78,90], but were not taken into account in the present study.

### Conclusions

This study investigated, for the first time, FD and CV slopes as indexes of central and peripheral muscle fatigue during isometric contractions in healthy humans. The significant correlation between FD and CV found at 60% MVC suggests that a mutual interaction between central and peripheral fatigue can arise during submaximal isometric contractions and may represent an adaptation to muscle fatigue.

Further studies are needed to confirm FD and CV as universally acceptable indexes of central and peripheral fatigue, and their roles in unraveling the impact of both of these aspects of fatigue in sport sciences and several diseases.

## Supporting Information

S1 File Raw data of sEMG acquisitions and subjects features. (XLSX)
